# First Onset of IgA Vasculitis and Nephritis Following COVID-19 Vaccination

**DOI:** 10.7759/cureus.42448

**Published:** 2023-07-25

**Authors:** Jesús Iván Martínez-Ortega, Felipe Perez-Hernandez, Ilse Fernández-Reyna, Nixma Eljure Lopez

**Affiliations:** 1 Department of Dermatology, Dermatological Institute of Jalisco “Dr. José Barba Rubio”, Zapopan, MEX; 2 Department of Internal Medicine, Hospital Regional de Alta Especialidad de la Peninsula de Yucatan, Merida, MEX; 3 Department of Dermatology, Centro Dermatologico de Yucatan, Mérida, MEX

**Keywords:** nephritis, vasculitis, covid-19, iga vasculitis, vaccine-induced vasculitis, iga nephritis, covid-19 vaccine

## Abstract

The article presents a case of a 32-year-old male who developed IgA vasculitis (IgAV) and IgA vasculitis nephritis (IgAVN) after receiving the second dose of the AstraZeneca COVID-19 vaccine. IgAVN can be a rare side effect of COVID-19 vaccines. Healthcare providers should be aware of this potential adverse event, and promptly recognize and manage it. However, the benefits of vaccination in reducing the morbidity and mortality associated with COVID-19 far outweigh the risks of this rare adverse event.

## Introduction

The COVID-19 pandemic has led to the rapid development of vaccines against the SARS-CoV-2 virus, with millions of people worldwide receiving the vaccines. While most individuals experience only mild local or systemic reactions to the vaccine, reports of dermatological side effects have been increasing. Cutaneous vasculitis, a rare skin reaction, has been reported in a small number of cases following COVID-19 vaccination [1]. It is currently unclear whether these reactions are causally related to the vaccine or represent a chance association. In this case report, we describe a patient who developed IgA vasculitis (IgAV) and IgA nephropathy following the administration of the AstraZeneca COVID-19 vaccine. We discuss the possible mechanisms underlying vaccine-induced vasculitis and the importance of monitoring and reporting these rare side effects to improve clinical management.

## Case presentation

A 32-year-old male came to the clinic complaining of a pruritic purpuric rash that developed three days after receiving the second dose of the AstraZeneca COVID-19 vaccine. He also had associated symptoms of fever, itching, and joint pain, which led to his admission to the emergency department. The patient's fever preceded the rash. He was not taking any medications at the time of presentation. He did not have arthralgia or arthritis, and there was no recent history of other vaccinations. Drug allergies were denied.

**Figure 1:**
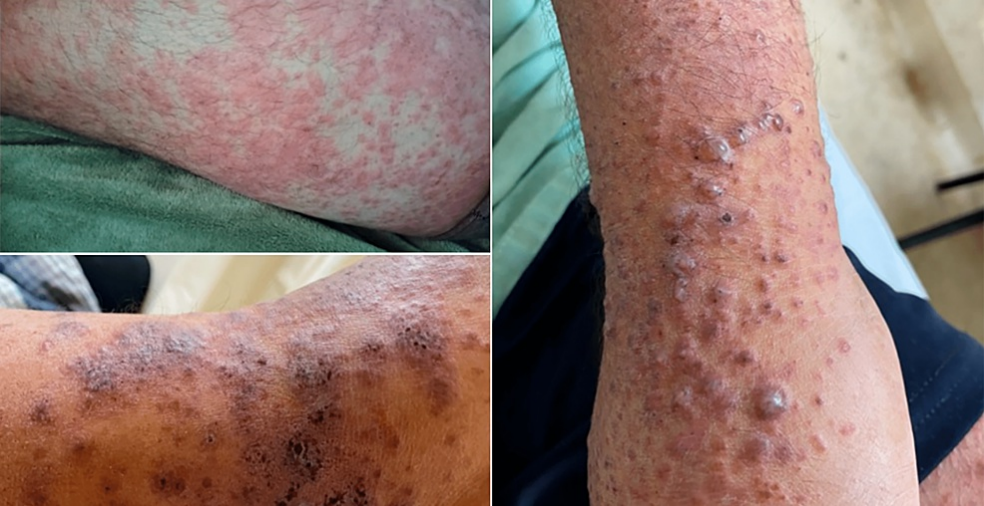
Skin lesions Erythematous-violaceous nodules on the upper and lower limbs.

A dermatosis affecting the upper extremities, abdomen, buttocks, and lower extremities was observed upon examination. It consisted of erythematous-violaceous nodules, some of which merged into plaques (Figure 1).

**Figure 2:**
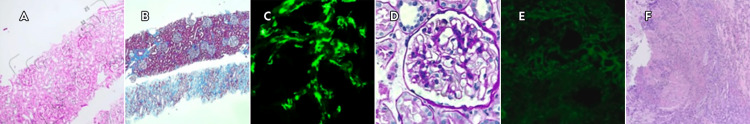
Skin biopsy (A) Hematoxylin and eosin staining: prominent, vacuolated podocytes without segmental lesions. (B) Masson's trichrome stain: interstitial fibrosis with tubular atrophy affecting 10% of the cortical surface, Grade I. (C) Direct immunofluorescence: diffuse and global positive staining for IgA with granular pattern in the mesangium and some segments of the glomerular basement membrane. (D) Periodic acid-Schiff stain: glomerulus with mesangial proliferation involving more than 50% of the glomerular tuft. (E) Skin immunofluorescence: negative staining for IgA, IgM, and IgG. (F) Skin histopathological examination with hematoxylin and eosin staining at 10x and 40x showing neutrophilic inflammatory infiltrate with abundant fragmented neutrophil nuclei and leukocytoclastic vasculitis.

The laboratory analysis showed leukocytosis (11,000/μL), elevated C-reactive protein (4.7 mg/dL), and an elevated erythrocyte sedimentation rate (46 mm/hr). Serology and immunology tests were normal (antinuclear antibody, antineutrophil cytoplasmic antibody, cryoglobulins, rheumatoid factor, C3, and C4). Polymerase chain reaction tests for SARS-CoV-2, Rickettsia, Leptospira, HIV, venereal disease research laboratory (VDRL), Epstein-Barr virus (EBV), hepatitis panel, and cultures were negative. Serum IgA levels were elevated (451 mg/dL). Imaging studies did not reveal any abnormalities. A skin biopsy was performed, and leukocytoclastic vasculitis was diagnosed (Figure 2).

Due to severity and progression, the patient was started on prednisone at a dose of 1 mg/kg/day and azathioprine at a dose of 2 mg/kg/day for three weeks, which resulted in almost complete improvement, and he was discharged.

One month later, he presented with hematospermia, hematuria, and new purpuric lesions. Urinalysis showed proteinuria of 150 mg/day and dysmorphic red blood cells. Renal and prostatic ultrasound were normal. A renal biopsy was performed, which confirmed IgA nephropathy (Figure 2). A new skin biopsy of a recent lesion was taken, once again showing leukocytoclastic vasculitis with negative immunofluorescence (Figure 2). He continued outpatient treatment with steroids and azathioprine. A complete resolution was achieved after three weeks.

## Discussion

IgAV and IgA vasculitis nephritis (IgAVN) are much less frequent in adult patients, and although their etiology is still uncertain, it is known that IgA is triggered by external factors related to immunopathogenic mechanisms, such as infections, vaccines, and drugs [2,3].

To the best of our knowledge, this is the first reported case of IgAVN associated with the COVID-19 adenovirus-based vaccine. Recently, Corrà et al. reported on 39 patients who developed cutaneous vasculitis following COVID-19 vaccination. Of those affected, 24 were women and 15 were men, with five cases being IgAV (12.8%), mostly related to mRNA vaccines, while 11 (28.2%) were associated with adenoviral-based vaccines. Specifically, 10 of those were related to the AstraZeneca vaccine, and two of the five cases of IgAV (40%) were associated with adenoviral-based vaccines. Sixteen patients (41%) experienced symptoms after the second dose. Only one of them presented biopsy-proven IgAVN following a recent influenza vaccination. In addition to the cases reported by Corrà et al., we also found two cases in the current literature of biopsy-proven IgAVN. These two cases of IgAVN were associated with the ChAdOx1 nCoV-19 vaccine [1,4,5].

Although there have been scarce reports of IgAVN associated with COVID-19 vaccines, including adenovirus-based, whole-inactivated virus, and RNA-based vaccines, the occurrence seems more related to host determinants than the type or nature of the immunization [1]. Furthermore, IgAV and IgAVN have been reported after the first, second, or third doses of the COVID-19 vaccine. As previously mentioned, the host determinant in IgAVN may be the hypoglycosylated IgA, which has a deficient hinge region in galactose that more easily binds to the mesangium and forms immune complexes that activate the immune system [2].

## Conclusions

In conclusion, while cutaneous vasculitis induced by the SARS-CoV-2 virus is rare, there have been increasing reports of skin reactions related to COVID-19 vaccination, particularly with the second dose and in younger individuals. Although the exact mechanism remains unclear, vaccines have been associated with various types of vasculitis, including IgAV and IgAVN. It is important to continue monitoring and reporting these rare side effects to better understand their potential causality.
